# The perceptions of adult psychiatric inpatients with major depressive disorder towards occupational therapy activity-based groups

**DOI:** 10.4102/sajpsychiatry.v27i0.1612

**Published:** 2021-02-26

**Authors:** Enos M. Ramano, Marianne de Beer, Johannes L. Roos

**Affiliations:** 1 Department of Occupational Therapy, Faculty of Health Sciences, University of Pretoria, Pretoria, South Africa; 2 Department of Psychiatry, Faculty Health Sciences, University of Pretoria, Pretoria, South Africa

**Keywords:** major depressive disorder, occupational therapy, activity-based groups, adult psychiatric inpatients, perceptions, occupational therapy groups

## Abstract

**Background:**

Occupational therapists have been using group therapy as their preferred treatment modality in mental healthcare since the origin of the profession. In private mental healthcare units, major depressive disorder (MDD) is the most common psychiatric disease. Occupational therapists use individual and group therapy to treat adult inpatients with MDD. Little is known about the perceptions and experiences of adult inpatients with MDD regarding occupational therapy activity-based groups.

**Aim:**

To describe the perceptions and experiences of adult psychiatric inpatients with MDD towards occupational therapy activity-based groups. This article reports on the perceptions of adult psychiatric inpatients with MDD, which formed part of a larger study.

**Setting:**

The study took place at two private general hospitals in Gauteng province, South Africa, each with a psychiatric ward.

**Methods:**

The researcher used a qualitative explorative descriptive design. Accessible participants were selected using convenience sampling. Only consenting participants took part in the study. Data were collected during focus group discussions. Data were thematically analysed.

**Results:**

Participants’ perceptions could be placed into one of four themes: (1) experience improved mood, (2) learned coping skills, (3) regained self-esteem and (4) becoming part of the solution to face life challenges.

**Conclusion:**

Activities that are unique to occupational therapy profession can benefit inpatients with MDD. This supports the profession’s historical beliefs, assumptions and foundations regarding therapeutic use of activities. According to these inpatients, group activities improved their overall mental health.

## Introduction

The practice of using activities for the treatment of mental health problems has lain dormant for decades according to the occupational therapy practice framework.^1^ Occupational therapists have been using group therapy as their preferred treatment modality in mental healthcare since the origin of the profession.^2,3,4^ As early as 1992, Polimeni-Walker, Wilson and Jewers^5^ found that inpatients viewed occupational therapy interventions as a means to reduce boredom and prevent them from ruminating about their problems. Long^6^ and Cole^7^ found that occupational therapy groups helped to reduce isolation by creating social bonds and strengthening individuals’ adaptive behaviour in a safe therapeutic environment.

In private mental healthcare units, major depressive disorder (MDD) is the most common psychiatric disease. Globally, MDD places a heavy burden on both individuals and society.^8,9^ Adult inpatients with MDD usually present with a depressed mood and reduced interest or pleasure in doing activities.^10,11^ An estimated 30% – 60% of individuals with MDDs have impaired social functioning even after they have achieved remission.^12,13^

In private acute psychiatric units, MDD is commonly treated with antidepressants, psychotherapy, interpersonal therapy^8,9,10,14^ and occupational therapy.^15,16,17,18^ Different types of occupational therapy groups are used in mental health, including functional groups, activity groups, task groups, social groups, life skills groups, psychoeducation groups, socioemotional groups and support groups.^1,6,18,19,20^

Sundsteigen, Eklund and Dahlin-Ivanoff^4^ explored patients’ experiences of groups in outpatient mental health services and found that occupational therapy groups assisted with personal growth, social change and acceptance of own responsibilities. Similarly, Lim, Morris and Craik^21^ concluded that occupational therapy groups afforded opportunities for socialisation, promoted creative expression, improved self-confidence and empowered individuals to practice a new skill in an environment offering support, relaxation and relief from boredom. Occupational therapy groups may also help patients to feel accepted, fostering a sense of belonging, relating to other people’s experiences, valuing themselves and recognising personal needs whilst constantly striving for occupational balance.^22,23^ Although there is much anecdotal evidence supporting the use of activity-based groups in community mental health, there seems to be a lack of rigorous scientific research to support the practice of activity-based groups.^24^

In this study, the researchers focused on adult inpatients’ perceptions and experiences of occupational therapy activity-based groups, where an activity was inherent in each group therapy session. The researchers could not find any research articles describing the perceptions and experiences of adult inpatients with MDD regarding occupational therapy activity-based groups. Thompson and Blair^25^ stated that occupational therapy activity-based groups have been criticised for existing to keep inpatients busy or to exploit free labour. In contrast, Lloyd and Williams^26^ argued that occupational therapists working in acute inpatient settings require further (1) acknowledgement, (2) reflection and (3) debate.

In private psychiatric units, occupational therapists form part of the multidisciplinary team responsible for treating inpatients with MDD. In this article, the researchers focused on the perceptions and experiences of adult inpatients with MDD towards occupational therapy activity-based groups. In the main study, inpatients with MDD received either a standard care occupational therapy group programme, with a combination of discussion groups or activity-based groups, or a standard care plus occupational therapy group programme that includes activities in each group therapy session. This qualitative study reports on the perceptions and experiences of participants who attended the standard care plus occupational therapy group programme, which comprised of activity-based group sessions.

## Methods

### Study design and setting

#### Design

This study followed a qualitative, explorative, descriptive design.^27,28,29,30^ The researchers used this design to seek new insight and clarification of adult psychiatric inpatients’ perceptions of occupational therapy activity-based groups.^28^ Social constructivism was used to construct knowledge about reality and not constructing reality itself.^31^ Thus, in this study, participants constructed their own multiple realities about the activity-based group intervention that they had attended, which included their own experiences and interaction with other participants.

#### Research setting

The study took place at two private general hospitals, referred to as Hospital A and Hospital B, in the Gauteng Province, South Africa, each with a psychiatric ward that had a 16-bed capacity for inpatients. The multi-professional psychiatric treatment team included psychiatrists, clinical psychologists, nursing staff, social workers and occupational therapists. As part of their intervention services, psychiatrists and social workers each led one group therapy session per week. The clinical psychologist had daily group therapy sessions. The occupational therapist presented daily activity-based group sessions.

#### The sample

The study population consisted of both male and female patients who were admitted to the psychiatric ward at Hospital A and Hospital B and met the inclusion criteria of the study. Inclusion criteria involved (1) the participants diagnosed with MDD (moderate to severe) with single or recurrent episode and (2) age groups ranged from 23 to 60 years as it was an adult psychiatric unit and a working age group. Ninety-seven adult psychiatric inpatients were recruited and consented to take part in the study. The study comprised 50 adult inpatients (25 from each hospital) who completed the occupational therapy activity-based programme, men (14%) and women (86%). Data were collected over a period of 4 months from March 2016 to June 2016. Participants were selected using a convenience sample approach^32,33^ who were available in the hospitals and admitted to the psychiatric ward.^32,34^ All participants consented to take part in the study.

#### Intervention programme

The group sessions in the occupational therapy activity-based group programme were graded from the building of interaction to giving each other positive feedback. The researcher classified the groups according to the activity-support continuum in which the main elements included tasks, social activities and communication.^35^ The researchers also ensured the following: (1) that there was an activity and (2) that there was socialisation and communication in each group therapy session.

The participants took part in the occupational therapy activity-based group sessions for 2 weeks and each session lasted 90 min. All participants received the same intervention in terms of content, intensity and duration of the group session and programme.^36^ When conducting groups, the two occupational therapists followed a group procedure based on the principles of Yalom in Beyers and Voster,^37^ Creek and Lougher,^38^ Finlay,^35^ Voster and de Beer^39^ and Yalom and Leszcz.^40^ Each group session had an initial phase, activity phase and finally a reflection and discussion phase. The contents of each session are shown in Table 1.

**TABLE 1:** Occupational therapy activity-based groups provided to inpatients with major depressive disorder in two private hospitals.^41^

Session	Group topic	Makeup of each group
1	Getting acquainted	A modified card game was played amongst adult psychiatric inpatients to know each other as they shared information about themselves.
2	Stress management	A stress ball was made by each adult psychiatric inpatient. This was followed by discussion on symptoms of stress and other coping skills.
3	Beadwork necklace	A necklace beadwork pattern was created by each adult psychiatric inpatient followed by discussion of the necklace.
4	Assertiveness game	Playing of assertiveness board game where adult psychiatric inpatients shared on ways to be assertive.
5	Relaxation therapy	An adapted relaxation technique was performed, followed by a discussion of other ways of relaxing.
6	Recreation	Adult psychiatric inpatients played fingerboard as warm-up activity followed by blokus game in pairs. This was followed by discussion of the games in comparison to their lives.
7	Creating collage	Each adult psychiatric inpatient made a collage using drawing, art and craft materials, which was followed by sharing of the meaning of collage, reflection and support.
8	Decorating gift box	Various art materials (paper, acrylic paints, pastels, charcoal) were used to create and decorate a gift box of the best time of their lives.
9	Feedback	Each adult psychiatric inpatient created a greeting card using a variety of art and craft materials. On completion of the card decoration, members wrote feedback to each other.

#### Data collection

Data were collected during focus group discussions^42,43^ held at the end of the program, as the tenth group session before being discharged from hospital. The first author posed a range of semi-structured open-ended questions, which were followed as a focus group discussion.^44^ The questions were always asked in the same sequence with some paraphrasing, clarifying questions and prompting from the first author. This allowed the researchers to receive multiple viewpoints about the intervention in a shorter period of time.^28^ Each focus group had between 5 and 11 participants. Each participant gave consent before taking part in the focus group.

The first author acted as moderator and facilitated each focus group discussion. Three focus groups were held at each hospital, six in total. Data saturation was reached because the same themes kept emerging during the latter interviews. The focus group interview guide is shown in Box 1.

BOX 1 Interview guide used during focus group discussions held at the end of an activity-based group therapy programme for inpatients with major depressive disorder.

1. What are your comments about the occupational therapy groups you attended?
2. What helped you the most during the occupational therapy groups?
3. Which occupational therapy group came out the strongest for you and why?
4. Which group touched where it had to touch and why?
5. In your opinion which occupational therapy group is rated the lowest and should be removed from the programme?
6. What do you think should be added to the occupational therapy group programme?
7. Any other information you feel is important to comment about?

#### Data analysis

Focus group discussions were recorded and transcribed verbatim.^33,45,46^ Transcripts were thematically analysed as guided by Braun and Clarke.^46^ The researchers scrutinised the texts to identify the categories^33,47^ and supporting quotations from the data.^48^ Even during the inductive coding, the researchers kept using a bottom-up approach whilst reading through the transcripts^49^ to try and answer the research question. As a social constructivist, the researcher used participants’ views to build broader themes. Themes were renamed and revisited in relation to their extracts and meanings for the researcher.^46^ The second author scrutinised the transcript and themes and discussed with the first authors. Three independent coders analysed the transcripts and formulated their own themes. Consensus regarding final themes was reached between the authors and three independent coders.

### Quality of the study

The researchers followed Lincoln and Guba’s^33^ five criteria for developing trustworthiness: credibility, dependability, confirmability, transferability and authenticity. The researcher took accurate, detailed notes.

Credibility was achieved through extensive discussions between the researchers, three external auditors (independent coders) and the use of literature. The three external auditors formulated their own themes from the data and met the researchers to agree on the final themes and to ensure interpretive agreement.^29,30^ Different perspectives about the themes ensured that the themes were realistic and richer.^50^ The researchers used data triangulation by using focus groups, field notes and observations.^28,31^ The researchers documented the process of continuously checking and rechecking data.

### Ethical consideration

The researchers ensured that non-consenting inpatients had the opportunity to participate in the occupational therapy activity-based groups in a non-prejudicial manner.^33^ Consenting participants who took part in the occupational therapy activity-based groups were thus afforded the opportunity to reflect on their treatment perception and experience, including the benefits. This type of practice seldom happens in daily clinical practice. Therefore, the principle of beneficence was implemented.

The University of Pretoria Research Committee and the University of Pretoria Faculty of Health Sciences Research Ethics Committee approved the study before commencing (ethics reference number: 226/2015). The management of the two hospitals gave permission for the study to take place in their hospitals. The researchers ensured that each participant voluntarily completed an ‘informed consent’ form.^43,51^ Participants were informed about the study and they knew they could withdraw at any time before or during participation. Consenting participants who took part in the occupational therapy activity-based groups shared their treatment perception and experience, including the benefits. This type of practice seldom happens in daily clinical practice. Therefore, the principle of beneficence was implemented. The researchers ensured that non-consenting inpatients had the opportunity to participate in the occupational therapy activity-based groups in a non-prejudicial manner.^33^

## Findings

**FIGURE 1:**
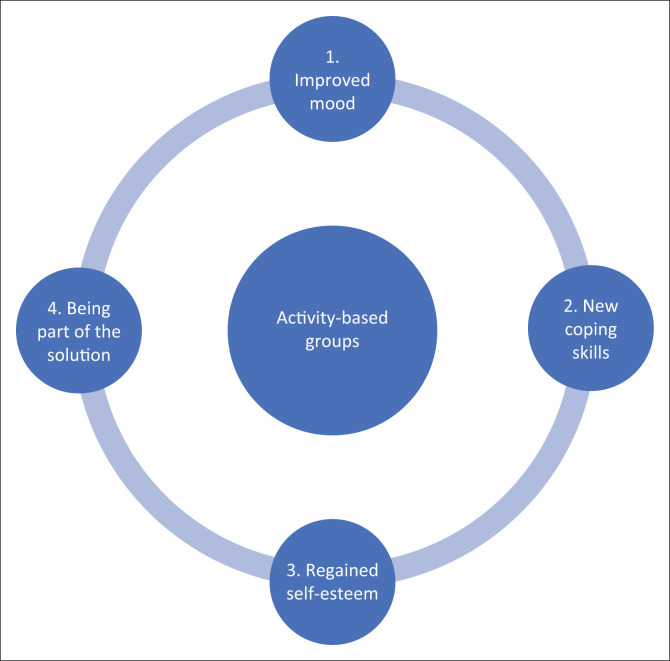
Inpatients’ perceptions of occupational therapy activity-based groups.

Four themes emerged from the participants’ perceptions about their experiences of the occupational therapy activity-based group programme: (1) experience improved mood, (2) learned coping skills, (3) regained self-esteem and (4) being part of the solution as shown in Figure 1.

### Theme 1: Experience improved mood

Most participants stated that they experienced improved mood:

‘… when you come back they [*other MHCUs*] find you in a bubbly mood and then they ask themselves … what happened and I tell them we attended [occupational therapy activity-based groups] and we did this and that ….’ (Participant 26, female, 28 years old)

‘… it [*occupational therapy activity-based group*] was very helpful because you wake-up in the morning and your mind is already occupied with why you are here and what makes you sad … and the moment you are called for a group session you find that in two minutes … you are already laughing ….’ (Participant 28, female, 28 years old)

Participants attributed their improved mood to various categories including (1) working together, (2) flow whilst engaging in activities and (3) task satisfaction. As the synthesis of the three categories lead to Theme 1, each category will be discussed separately.

#### Working together

Participants mentioned that working together elicited happiness and laughter, during and after occupational therapy activity-based groups:

‘We were working together as a group and writing good messages to each other … my spirit was lifted.’ [*card making and feedback*] (Participant 2, female, 48 years old)

‘We worked together and had fun to ease our mind and body.’ [*assertiveness game*] (Participant 16, female, 48 years old)

#### Flow whilst engaging in activities

The participants experienced flow whilst engaging in tangible activities which facilitated change and growth. The following are some of the participants’ statements, attesting to the idea that they experienced just the right challenge whilst engaging in activities.

‘…I was very down, very emotional that day, but being in the fingerboard … it released my mind where I was and I ended up being happy and laughing….’ (Participant 21, female, 29 years old)

‘…making cards for me was very interesting and I could focus and not think about my problems.’ (Participant 15, female, 43 years old)

#### Task satisfaction after completing the task

The participants experienced task satisfaction after successfully completing the tasks. This was supported by some of the participants:

‘…I felt complete after taking part in this group [*beadwork*] that I can still do something beautiful for myself [*completed the task*].’ (Participant 12, female, 33 years old)

‘I am happy because I did beads which I never did before.’ (Participant 27, female, 33 years old)

### Theme 2: Learned coping skills

The participants emphasised that the activity-based group therapy sessions helped them to learn coping mechanisms for their individual stressors. Participating in activity-based groups enabled three modes of learning including learning coping skills, learning new activity skills and learning about the use of free time. The benefits of learning through participation were elaborated as follows:

‘…when we were busy – like doing different things…learning this and that… it was like a real empowering workshop….’ (Participant 45, female, 32 years old)

‘… I learned different skills. I could do things I never thought I could do.’ (Participant 21, female, 49 years old)

#### Empowered to handle situations

The participants reported that they felt empowered to handle their situations:

‘…realized that I have been selling myself in a wrong way because I was never able to say No and if ever I said No – I felt guilty somehow and would try to make up for that. So – yah it really helped me….’ (Participant 19, female, 59 years old)

‘…Creating the stress ball and using it because even in there in the ward when I was thinking and somehow something just came in which hurts me, I would just take it and squeeze it, concentrate on it and then it distracts me….’ (Participant 20, female, 38 years old)

#### Learning new activity skills

The participants were excited that they discovered new activity skills, which they were not aware they could do:

‘I did not know my creativity until I did beads necklace. It was relaxing and I never had time to think about my problems.’ (Participant 46, female, 25 years old)

‘I did things that I don’t know with my hands…it brought out the creativity in me… I started things from scratch.’ (Participant 48, female, 24 years old)

#### Learned the use of free time

The participants reported learning how to constructively use their leisure time:

‘…It is something that you can do in your spare time, rather than spend your time worrying and sleeping….’ (Participant 3, male, 35 years old)

‘I learned that I can do something productive during my spare time….’ (Participant 9, female, 40 years old)

### Theme 3: Regained self-esteem

The participants reported gaining self-confidence by participating in concrete tangible activities:

‘I could see that I am talented, have a skill, I can overcome a challenge and I can be happy.’ [*card making and feedback*] (Participant 1, male, 43 years old)

‘It gave me hope and a sense of belief in myself that I can do it.’ [*collage*] (Participant 13, female, 25 years old)

#### Mastered skills to perform activities

Participants successfully created a meaningful end-product, which according to them improved their self-confidence:

‘…The beads, some of them had alphabets so you could actually create something that has a meaning…and you can master.’ (Participant 48, female, 24 years old)

‘…you realize that you did something very nice and something very meaningful. I mean something that you eventually mastered for yourself….’ (Participant 47, female, 39 years old)

#### Inspirational positive feedback

Participants appreciated inspirational positive feedback from others, which improved their self-esteem:

‘Being seen as a good person and it made me to feel good about myself. It was a mind opener about myself and it worked on my confidence ….’ [*card making and feedback*] (Participant 44, female, 32 years old)

‘I…gathered more of what was hidden inside and what I never knew was there….’ (Participant 35, female, 37 years old)

### Theme 4: Being part of the solution

The participants mentioned that the collage activity helped them realise that they had to face their problems and be part of their solutions:

‘I believe everything happens for a reason and that I should face reality. The past is there and we need to close that chapter by creating good new memories.’ (Participant 29, female, 38 years old)

‘Being part of the group taught me to face my challenges that I was having…I realised that I have to bring solutions to my problems.’ (Participant 27, female, 33 years old)

#### Gaining insight

Most participants reported gaining insight to their problems as they shared with other participants. Listening to others’ problems helped them put their own problems into perspective. This was shared by two participants who said:

‘You don’t have to be weak, I have to stand up and face reality and deal with it. If you want to achieve goals in your life you must start with the old matters and deal with them, then focus on the new ones, then you will see progress.’ (Participant 35, female, 37 years old)

‘It reminded me of my best time that I can still do well in life. Otherwise, the program is brilliant…it touches different parts of life…I realized that other people have worse problems than myself.’ (Participant 49, female, 24 years old)

#### Courage to face problems

Participants felt that the activity-based group therapy sessions provided a supportive environment, giving them the courage to face their problems. This is shared by participants who said:

‘It [*the activity-based group*] gave me hope and a sense of belief in myself that I can do it.’ (Participant 14, female, 32 years old)

‘It reminded me of the achievements in my life and gave me hope that I can do it again.’ (Participant 19, female, 59 years old)

## Discussion

In this study, the authors used focus groups to explore the perceptions of adult psychiatric inpatients with MDD towards occupational therapy activity-based groups.^52^ Our participants were on average 37.3 years old, close to the mean age of onset for major depression, which is about 40 years.^10^ The participants also mirrored the general trend of more women than men who are diagnosed with MDD.^10^ From the patients’ perspectives, occupational therapy activity-based groups resulted in favourable outcomes, including improving mood, learning coping skills, improving self-esteem and being part of the solution.

Polimeni-Walker, Wilson and Jewers^5^ reported that under-stimulation slows the process of MDD recovery. Inpatients with MDD who participate in occupational therapy activity-based groups are more stimulated and feel happier. Experiencing improved mood happens when there is flow during occupational engagement and when the activities are challenging enough.^4^ Mood improves as patients experience happiness, inner enjoyment and challenge. To achieve flow, the individuals must be engaged in the ‘just right challenge’.^19,38^ As the inpatients with MDD were immersed in challenging activities, their mood improved. They felt relaxed and calmer and became rational in dealing with their problems whilst working together.

Working together whilst engaged in meaningful activities allowed inpatients to develop feelings of belonging, sharing and opening up as part of the healing process. This is in line with Wilcock’s notion of ‘doing, being, becoming and belonging’, as she believed that people, through occupation, are in a ‘constant state of becoming different’.^53^ Whilst working together, participants showed interest and were motivated to participate and work together in a group.^54^ Working together resulted in fellowship and creation of relationships.^55^ For example, one participant commented: ‘…we got to know each other, laugh together, doing things together….’ Participation in this context may be understood as a willingness to exert selfless effort to contribute to the well-being of others,^56^ praising effort only if it is altruistic in nature. Cooperation plays an important role in activity participation as ‘each member is expected to consider himself an integral part of the whole and to play an appropriate role towards achieving the good of all’.^56^

Successfully completing the tasks through cooperation also improved the mood of participants who had better energy levels, were more interested and felt excited after completing their tasks with pride.^57^ In this programme, the available activities provided an opportunity for personal satisfaction.^19,58^ According to Reid,^59^ engaging in meaningful occupation leads to feelings of satisfaction and pleasure. In this study, participants felt happiness, pleasure, laughter and inner fulfilment brought about through task satisfaction. According to Steger and Kashdan,^60^ people with MDD experience greater satisfaction and meaning when their need to belong is met, which was achieved during participation in this study.

During the activity sessions, participants shared ideas and ways of coping with different situations in their lives. Completing activities allowed inpatients to learn life skills, which equipped them with effective and healthy coping skills as they imparted knowledge and assisted each other in dealing with challenging life situations. This tallies with the finding by Sundsteigen, Eklund and Dahlin-Ivanoff^4^ that participants will find alternative ways to better health through learning in the group. Finlay adds that activities aim to develop skills,^61^ which may be used in the daily lives of participants. In this study, participants experienced healing and wellness whilst doing, and they also learned a valuable skill that could be performed in their lives. Lim, Morris and Craik^21^ reported that most of their participants found that occupational therapy interventions were helpful in learning a new skill.

New skills can also be learned during free time. Using free time helps to satisfy individual needs that are not met by either self-care or work.^19^ Some of the group activities promoted the constructive use of free time whilst learning new activity skills. After discharge, participants may be able to keep doing free time activities helping them to experience a healthier, balanced lifestyle.^62^

Engaging in meaningful activity is essential to a person’s well-being. Activities in occupational therapy afford inpatients with MDD an opportunity to improve self-esteem.^38^ Inpatients develop confidence when they are admired and acknowledged by others as was observed in this study.

Purposeful activity and meaningful activity are core skills of the occupational therapy profession.^38^ The occupational therapy activity-based group brought some meaning to the participants as supported by one statement, ‘…you realize that you did something very nice and something very meaningful. I mean something for yourself….’ (Participant 47, female, 39 years old). Purposeful activity provides an opportunity for an individual to achieve mastery, thus gaining a sense of inner assurance and competence.^19,38^

## Limitation of the study

This study focused on adult psychiatric inpatients with MDD in a private psychiatric unit in South Africa. Inpatients who use private health facilities are those who can afford medical insurance. The researchers did not test this intervention in a public health setting, so the findings are not generalisable to all South African psychiatric inpatients with MDD. The study used data that were collected for 2 weeks during the acute phase of inpatients with MDD. The researchers did not assess the influence of other treatments offered during the period; it is likely that progress made during treatment is cumulative. The researchers did not follow-up on patients after discharge including the maintenance of gains.

The researchers assessed specific activity-based groups, but these should not be seen as a prescriptive procedure or a ‘blue print’ but rather as a set of steps.

The researchers did not explore the influence of the occupational therapist or the perceptions of the inpatients regarding the occupational therapist. From the participants’ comments, it would seem that the therapeutic relationship played a vital role in the healing process.

## Conclusion

Historically, occupational therapists have always recognised the value of doing activities. The researchers showed that inpatients perceived that activity groups were valuable. According to the inpatients, they felt empowered, more confident and better able to deal with their problems. The activity-based groups also provided an opportunity to work together, to share their experiences and generally had better therapeutic outcomes. Our findings encourage and support using activities as part of occupational therapy groups.
